# Wire or coated balloon? Searching for an optimal source for intravascular brachytherapy with *β* emitters using ${}^{32}P$ as an example

**DOI:** 10.1120/jacmp.v4i1.2542

**Published:** 2003-01-01

**Authors:** J. Lehmann, C. R. King

**Affiliations:** ^1^ Department of Radiation Oncology Stanford University School of Medicine 300 Pasteur Drive, Room A‐055 Stanford California 94305‐5304

**Keywords:** intravascular brachytherapy, in‐stent restenosis, beta emitters, Monte Carlo

## Abstract

This study identifies basic dosimetric differences between two designs for intravascular brachytherapy (IVBT) in current clinical practice and ongoing trials and their clinical implications within beta emitting systems using P‐32 as an example. The two designs are (i) the wire‐type source, where the radioactive source material is confined to a wirelike structure within the vessel lumen, and (ii) the balloon‐surface source, where the radioactive source material is distributed over a surface area (balloon‐wall) which is brought in close proximity with the vessel wall. Using Monte Carlo simulations with the EGS4 code, the target coverage, the influence of centering errors, and the perturbation of the dose distribution caused by metallic stents have been compared. The radial dose fall‐off in the target region was found to be steeper for balloon surface systems compared with wire systems. The inner lumen wall dose for a balloon surface source was 25% higher than that for a wirelike source (2.5 mm vessel diameter). However, the comparably shallower fall‐off from wire‐type systems is very sensitive to centering uncertainties. A 0.5 mm displacement, for example, will cause the dose to change by a factor of 2 at the inner vessel wall and by a factor of 1.8 at the prescription point. It is shown that the interference from metallic stents is more significant for wire‐type systems than it is for balloon‐surface‐type systems, where double the dose variation beyond the stent at the radial prescription distance may occur. Centering uncertainties dominate the dose perturbation effects for wire‐type systems. Balloon‐surface‐type designs show a more predictable dose distribution that features, however, a higher inner vessel surface dose. Since a direct clinical comparison of systems of both types is not likely, these findings should be considered when interpreting clinical results from treatments with either type of source and, possibly, for future source design. © *2003 American College of Medical Physics.*

PACS number(s): 87.53.–j, 87.90.+y

## INTRODUCTION

The use of ionizing radiation following percutaneous transluminal coronary angioplasty has been shown in several randomized trials to inhibit restenosis of the vessel, and it is now an accepted therapeutic intervention for in‐stent restenosis while it remains investigational for *de novo* lesions.^1^ Currently, there are three FDA‐approved devices for clinical use: the Beta‐Cath™ system from Novoste (Novoste Corporation, Norcross, GA), the Galileo™ system from Guidant (Guidant Corporation, Houston, TX), and the Checkmate™ system from Cordis (Cordis Corporation, Miami, FL). These are all so‐called wire‐type systems in that the radioactive source is distributed along a wirelike structure (or linear source train) that is more or less centered within the vessel lumen. A competing system is a balloon‐surface‐type design where the radioactive material is distributed evenly over the surface of a balloon that is brought into close proximity with the vessel wall. These were used in clinical trials with the RDX system [Endologix (former Radiance Medical Systems). Irvine, CA], and other balloon‐surface‐type systems are presently under development.^1^

The many competing systems in use or being developed possess several important differences: (a) beta versus gamma radiation, (b) the energy spectrum of the isotope used: Sr‐90, Ir‐192, P‐32, I‐125, etc., (c) the use of centering devices, and (d) the geometrical design (i.e., wire‐type versus balloon‐surface‐type). While there are several studies comparing beta versus gamma systems (e.g., Amols *et al.* ^2^ and Li *et al.* ^3^), there are few studies which describe the implications of different geometrical designs. A dosimetric analysis is essential because a randomized study that directly compares these different systems is highly unlikely. Going from wire‐type sources to balloon‐surface type sources where the radioactive material is in almost direct contact with the lumen wall changes the irradiation geometry significantly. Two potential effects will arise: one is the influence of obstructing materials such as metallic stents on the dose delivered beyond the stent, and the other is the error in target dose due to centering errors.

This study explores the dosimetric differences between these two designs, wire‐type and balloon‐surface type, in P‐32 beta emitting systems that might bear significant clinical implications and which should be recognized in anticipation of clinical outcomes.

## METHODS

For this investigation the Monte Carlo method was chosen as it allows the analysis of dose distributions for user‐defined geometries with a high degree of accuracy. The Monte Carlo system employed for this study is EGS4,^4^ which is widely used and has been validated in many studies. Here, we modeled two distinct source geometries: the “wire‐type” and the “balloon‐surface‐type” designs with a modified version of the dosrz user code. We determined the dose to water for points around the sources in a fine grid (0.2 mm in the radial and in the longitudinal dimension), assuming either no obstruction or a specific obstruction such as a stent. The simulation parameters including the grid size have been based on previous work^5^ and on prestudy investigations. Our complete simulation system has been validated in comparison with measurements and the simulation of others. This was done for complete Guidant source wire, including housing, and has been reported previously.^6^ Other authors have also simulated this source and established agreement between measurement and Monte Carlo simulation.^7^–^9^

**Figure 1:**
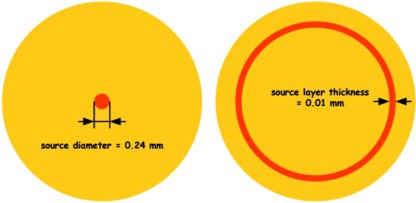
(Color) Schematic of the radial source dimensions for the wire‐type source (left) and the balloon‐surface‐type source (right). Not to scale.

High‐Z materials found in current stents as well as present in vessel calcification cause significant dose perturbations of beta radiation by absorption and backscatter, whereas it is negligible for gamma radiation.^3^ Consequently, this study focused on the beta emitting systems and specifically using the radioisotope P‐32. Its conclusions are applicable to all beta emitters. In order for our conclusions to be generalized the sources were modeled without any manufacturer's specific housing materials. However, the dimensions of the source were chosen similar to the GALILEO system (wire‐type) and the 2.5 mm RDX system (balloon‐surface‐type), two systems that use P‐32. The wire‐type source has a diameter of 0.24 mm (Fig. 1). The thickness of the source layer in the balloon‐surface‐type system has been set to 0.01 mm. No other parts of the balloon were simulated, assuming all water. This assumption is not unrealistic since the outer balloon layer is very thin (i.e., about 0.01 mm for the RDX device) and similar to water with respect to radiation transport (the exact composition and size are proprietary in case of the RDX device). Leaving out specific materials preserves the general approach of the work.

The dose was normalized to a prescription point that is specified with respect to anatomy rather than the source, namely 1 mm into the vessel wall. While there is still debate over this issue, this choice allows a straightforward comparison between systems and also reflects the current accepted clinical practice for the P‐32 systems.

**Figure 2:**
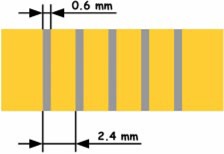
(Color) Simple stent geometry (ring bands) with a 25% metal surface area.

Initially, the wire‐type source is assumed to be perfectly centered within the vessel lumen. However, since this is rarely the case in practice even with self‐centering devices, we examined the dosimetric implications of deviations from perfect centering. Balloon‐surface‐type systems, on the other hand, are in direct or very close contact with the vessel wall and as such source deviations from the target tissue are small enough to be negligible. IVBT is often used in the presence of a metallic stent. (In the U.S. it is at present clinically approved only for in‐stent restenosis.) Current stents that can be found in arteries range in their metallic surface area (percentage of stent surface area covered with metal) from about 10% to above 30%, depending on stent design and expansion (vessel size).^10^,^11^ For the Monte Carlo simulations a simple stent geometry (Fig. 2) with a metallic surface area of 25% has been assumed. While this simple ring band design was initially based on the specifics of the Monte Carlo user code employed, it also shows the extreme case of a stent influence and how it is different for either source design.

## RESULTS

First, a comparison between the two types of IVBT designs has been made in the absence of a stent. It focused on the dosimetric profile from the two geometries as well as the consequences of centering errors within the vessel lumen. Second, a comparison has been made between the two designs in the presence of a metallic stent.

### A. Comparison between wire and balloon‐surface‐type sources in the absence of a metallic stent

**Figure 3:**
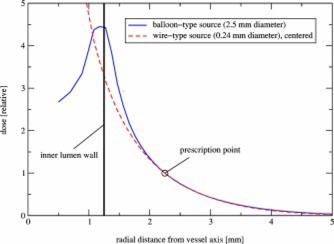
(Color) Radial dose fall‐off in the center plane of a radioactive coated balloon (2.5 mm diameter) compared to an ideally centered wirelike source (0.24 mm diameter) of the same P‐32 isotope. For both, the dose is prescribed 1 mm into the vessel wall.

Figure 3 shows the radial dose fall‐off at mid‐plane for a balloon‐surface‐type source (2.5 mm diameter, source thickness 0.01 mm) compared to a wire‐type source (0.24 mm diameter) of the same isotope P‐32. The inner lumen wall coincides with the outer balloon dimension. In both cases the dose is prescribed at 1 mm into the vessel wall. The dose fall‐off is shallower for the wire‐type source in the first millimeter of vessel wall. However, the dose fall‐off holds true only for a perfectly centered source wire.

**Figure 4:**
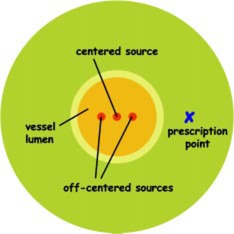
(Color) Schematic of ±0.5 mm shift of a 0.24 mm wire within a 2.5 mm vessel. Proportions are shown to scale.

While the position of a balloon‐type source is fixed relative to the vessel wall because of direct contact, a wire‐type source's position depends upon centering and inherently has a certain degree of uncertainty. In a “noncentered” wire system the thickness of the catheter determines the possible positional change of the wire. Thicker catheters hold the source more accurately centered but they also block more of the blood flow and are more difficult to maneuver. Even with a “centered system,” deviations from perfect centering exist, especially near vessel bends. We model errors in wire centering as shown in Fig. 4, where shifts are illustrated in a drawing to scale.

**Figure 5:**
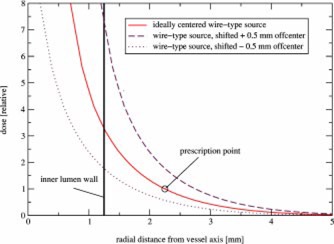
(Color) Radial dose fall‐off in the center plane of a wirelike P‐32 source of 0.24 mm diameter in a 2.5 mm diameter vessel (see also An ideally centered wire is compared with wires that are off‐centered by −0.5 mm (displaced away from the wall by 0.5 mm) and +0.5 mm (displaced closer to the wall by 0.5 mm). These shifts are shown in Fig.

Figure 5 shows the radial dose fall‐off for wires that were displaced from the center position with shifts of 0.5 mm of the wire position towards and away from the vessel wall. The dose at the prescription point increased by about 80% and at the vessel wall by more than 100% for the 0.5 mm shift towards the vessel wall. Shifting the wire 0.5 mm away from the wall caused a 40% drop of the dose at the prescription point and a 45% drop at the vessel wall. Even for a smaller shift of just 0.2 mm (not shown in the diagram), the dose increased by 25% at the prescription point and by 33% at the vessel wall for a shift towards the vessel wall, and decreased by about 20% for a shift away from the wall.

### B. Comparison between wire and balloon‐type sources in the presence of a metallic stent

**Figure 6:**
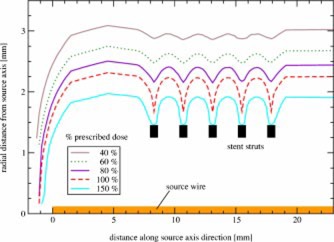
(Color) Dose distribution around a P‐32 source wire within a metallic stent. The dose has been normalized to 1 mm beyond the vessel wall (1.25 mm radius) in an unobstructed area.

**Figure 7:**
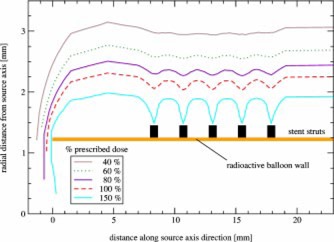
(Color) Dose distribution around a 2.5 mm diameter P‐32 balloon‐surface source within a metallic stent. The dose has been normalized to 1 mm beyond the balloon wall in an unobstructed area.

For the comparison of balloon‐and wire‐type sources a simple stent (Fig. 2) has been simulated around the 2.5 mm balloon‐surface source and the 0.24 mm wirelike source. Figures 6 and 7 show the dose distributions for either source with a stent in place.

**Figure 8:**
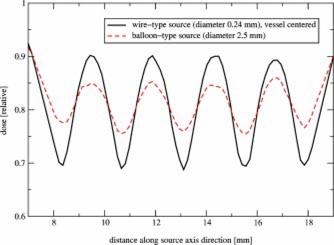
(Color) Dose at the prescription distance along the source axis for a stent‐obstructed wire‐type and balloon‐surface‐type sources. The dose has been normalized to the dose for the same source with no stent in place, making the displayed relative dose equal to the dose perturbation factor (DPF) as defined by Li *et al.* ^3^

It is apparent that a greater dose perturbation is present for the wire than for the balloon, as can be seen for the 100% isodose line. To quantify this effect, the doses at the prescription distance along the source axis direction were plotted in Fig. 8. The dose was here normalized to the dose for the same source with no stent in place. This is equivalent to the dose perturbation factor (DPF) introduced by Li *et al.* ^3^ that is defined as the ratio of doses with and without an interfering stent. For the balloon‐surface source the dose varied between 0.75 and 0.86 of the dose without a stent (a 7% variation about the mean dose, the mean dose within the stented area being 0.81 for either balloon or wire source). For the wire the variation was between 0.69 and 0.91 (a 15% variation about the mean dose). These numbers will, of course, vary for different stent geometries and specifications. However, they show that the dose distribution around a balloon‐surface source is significantly less affected by a stent (by approximately half as much), while the average DPFs are similar for wire and balloon‐type source.

## DISCUSSION AND CONCLUSION

Wire‐type systems show a shallower radial dose fall‐off over the target region than balloon‐surface‐type systems. Therefore, the latter display a higher inner vessel wall dose for equal prescription dose. This presumable advantage of the wire‐type system is actually only true for a perfectly centered wire within a perfectly round vessel. Small and expected deviations from this ideal situation can lead to dramatic changes in the dose distribution, by as much as a factor of 2, as we have shown. The numbers found here for the 0.5 mm shift agree with those of Chibani and Li^12^ and Sehgal *et al.* ^13^ who investigated the source offset effect for several commercial IVBT sources using the MCNP Monte Carlo code.

While the balloon‐type sources have a slightly steeper dose‐fall‐off over the target region, their position is fixed relative to the wall and the deviations present in wire‐type systems are not to be expected. Balloon‐type sources are likely to either adapt their shape to the vessel wall or to expand it slightly, in either case achieving a good balloon‐vessel wall contact (assuming an appropriately chosen balloon diameter). Dose deviations analogous to those seen in wire‐type systems will exist of course if a balloon‐type source is chosen that is too small with respect to the vessel diameter. Choosing a balloon that is too large would not affect the dosimetry; however, it might have other adverse effects such as over‐stretching of the vessel and thereby causing additional injury. In general it should be noted that the surface‐coated balloons are only expanded to bring the radioactive material in close contact with the vessel wall. No injury, as in the balloon dilatation, should be done to the vessel, because such injury would be a potential cause of additional neointima growth and the length of radioactive coating would not be sufficient to deliver an adequate dose to newly injured vessel segment. It is therefore essential to observe the manufacturer suggested inflation pressure for coated balloons.

With stents in place, balloon‐surface sources show a significantly smaller dose disturbance than wire‐type sources, about half as much. In our model a stent caused about a 7% dose perturbation about the mean dose, which is consistent with that actually measured experimentally,^14^ while the wire caused about a 15% dose perturbation about the mean dose. This difference can be explained by the proximity of the source to the stent that causes a smaller “shadow” effect. Detailed results for commercial source wires have been published by Ye *et al.* ^15^ and Fan *et al.* ^16^ An additional aspect is that guide wire interferences for wirelike sources, as described by Li and Shih,^17^ will play a different and much smaller role for balloon surface sources, since the wire is not located between source and target.

It seems reasonable to expect that such significant dose perturbations will bear clinical consequences. A dose‐response has been clinically proven for example in such trials as the Gamma I trial,^18^ where doses from 8 to 14 Gy resulted in restenosis rates of 58% to 20%, respectively. The Geneva II trials^19^ similarly showed restenosis rates of 28% to 8% for doses ranging from 9 to 18 Gy. Lower doses might not only be subtherapeutic but might paradoxically stimulate neo‐intimal proliferation,^20^,^21^ while higher doses might be associated with increased morbidity rates such as aneurysm formation and thrombosis.^22^ The issues surrounding the centering debate have been clearly summarized by Raizner.^23^

While current trials employing balloon‐type designs such as the BRITE II study using the RDX system from Radiance need time for the data to mature, interpreting their outcomes will require an understanding of the intrinsic dosimetric differences between them and the current wire‐type systems. Whether these differences translate into clinically meaningful consequences remains to be seen. It is reasonable to assume for the time being that dose perturbations as large as have been shown to exist might result in areas that are either underdosed or overdosed to a clinically meaningful degree and resulting in either restenotic areas or potential long‐term vessel damage.

## References

[1] Vascular Brachytherapy, 3rd ed., edited by Waksman R. (Futura, Armonk, NY, 2002).

[2] H. I. Amols et al., “Dosimetric considerations for catheter based beta and gamma emitters in the therapy of neointimal hyperplasia in human coronary arteries,” Int. J. Radiat. Oncol., Biol., Phys. 36, 913–921 (1996). 8960521 10.1016/s0360-3016(96)00301-x

[3] X. A. Li , R. Wang , C. Yu , and M. Suntharalingam , “Beta versus Gamma for catheter‐based intravascular brachytherapy: dosimetric perspectives in the presence of metallic stents and calcified plaques,” Int. J. Radiat. Oncol., Biol., Phys. 46, 1043–1049 (2000). 10705028 10.1016/s0360-3016(99)00457-5

[4] W. R. Nelson , H. Hirayama , and D. W. O. Rogers , “The EGS4 code system,” Report No. SLAC‐265, Stanford Linear Accelerator Center, Stanford, CA (1985).

[5] M. Lauterbach , J. Lehmann , and U. F. Rosenow , “Exploration of the Monte Carlo code EGS4 for the simulation of plane‐parallel electron chambers,” Z. Med. Phys. 9 (1), 38–46 (1999).

[6] J. Lehmann , K. M. Forster , and C.‐M. Ma , “Monte Carlo Investigation of Typical and Potential Sources for Endovascular Brachytherapy,” Proceedings of the XIII International Conference on the Use of Computers in Radiation Therapy (ICCR), Heidelberg, 2000 (Springer, Berlin, 2000), p. 492.

[7] F. A. Mourtada , C. G. Soares , S. M. Seltzer , and S. H. Lott , “Dosimetry characterization of 32P catheter‐based vascular brachytherapy source wire,” Med. Phys. 27, 1770–6 (2000). 10984223 10.1118/1.1286551

[8] R. Wang and X. A. Li , “Monte Carlo characterization of a 32P source for intravascular brachytherapy,” Med. Phys. 28, 1776–85 (2001). 11548949 10.1118/1.1388222

[9] T. D. Bohm , F. A. Mourtada , and R. K. Das , “Dose rate table for a ^32^P intravascular brachytherapy source from Monte Carlo calculations,” Med. Phys. 28, 1770–5 (2001). 11548948 10.1118/1.1384459

[10] “Interventional Vascular Product Guide,” edited by Leon M. and Mintz G. (Martin Dunitz Ltd, London, 1999).

[11] “Handbook of Coronary Stents,” edited by Serruys P. W. and Kutryk M. (Martin Dunitz Ltd, London, 1998).

[12] O. Chibani and X. A. Li , “Dosimetric effects of source‐offset in intravascular brachytherapy,” Med. Phys. 29, 530–537 (2002). 11991124 10.1118/1.1461373

[13] V. Sehgal , Z. Li , J. R. Palta , and W. E. Bolch , “Dosimetric effect of source centering and residual plaque for beta‐emitting catheter based intravascular brachytherapy sources,” Med. Phys. 28, 2162–71 (2001). 11695779 10.1118/1.1406520

[14] H. I. Amols , F. Trichter , and J. Weinberger , “Intracoronary radiation for prevention of restenosis: dose perturbation caused by stents,” Circulation 98, 2024–2029 (1998). 9808600 10.1161/01.cir.98.19.2024

[15] S. J. Ye , X. A. Li , J. R. Zimmer , J. C. Chu , and C. K. Choi , “Dosimetric perturbations of linear array of beta‐emitter seeds and metallic stent in intravascular brachytherapy,” Med. Phys. 27, 374–80 (2000). 10718142 10.1118/1.598841

[16] P. Fan , S. T. Chiu‐Tsao , N. S. Patel , A. Shih , K. Ravi , W. Sherman , H. S. Tsao , J. Pisch , and L. B. Harrison , “Effect of stent on radiation dosimetry in an in‐stent restenosis model,” Cardiovasc. Radiat. Med, 2, 18–25 (2000). 11068251

[17] X. A. Li and R. Shih , “Dose effects of guide wires for catheter‐based intravascular brachytherapy,” Int. J. Radiat. Oncol., Biol., Phys. 51, 1103–10 (2001). 11704335 10.1016/s0360-3016(01)01763-1

[18] P. Tripuraneni , “Gamma radiation to treat in‐stent restenosis: a dose response relationship,” International American Heart Assoc. annual. meeting, New Orleans, LA (1999).

[19] V. Verin et al., “Geneva dose finding study results update,” International Tenth Trans Catheter therapeutics meeting, Washington, DC (2000).

[20] J. Weinberger et al., “Intracoronary irradiation: dose response for the prevention of restenosis in swine,” Int. J. Radiat. Oncol., Biol., Phys. 36, 767–775 (1996). 8960502 10.1016/s0360-3016(96)00294-5

[21] J. Hausleiter et al., “Increased stenosis formation after low‐dose radiation therapy in balloon‐injured coronary arteries,” Circulation 100 (Suppl.), 74 (1999).

[22] Y. Vodovotz et al., “Effects of intracoronary radiation on thrombosis after balloon overstretch injury in the porcine model,” Circulation 100, 2527–2533 (1999). 10604891 10.1161/01.cir.100.25.2527

[23] A. E. Raizner , “The centering debate: the importance of centering in endovascular brachytherapy,” Vasc. Radiother Monitor 2, 3–10 (1999).

